# A GC-MC analysis of chemical compounds and identification of the antibacterial characteristics of the essential oil of two species exclusive to Iranian habitats: New chemotypes

**DOI:** 10.1371/journal.pone.0273987

**Published:** 2022-10-06

**Authors:** Mansureh Ghavam

**Affiliations:** Department of Range and Watershed Management, Faculty of Natural Resources and Earth Sciences, University of Kashan, Kashan, Iran; Universidad Autonoma de Chihuahua, MEXICO

## Abstract

**Background:**

The diversity found in the chemical compounds of a single species in different regions results in different biologic characteristics which can be considered as a strong source for identifying new chemotypes. *Hymenocrater incanus* Bunge and *Dracocephalum kotschyi* Boiss. are exclusive species of the Lamiaceae family which grow in the western and central habitats of Iran. This study was designed and carried out to determine the yield, identify the chemical compounds, and evaluate the antimicrobial characteristics of the essential oil (EO) of these two species in Iran for the first time.

**Methods:**

The flowering twigs of the species *D*. *kotschyi* and *H*. *incanus* were collected from the villages of Totmach and Kamu in Isfahan province respectively, in May 2019. The EO of these plants was extracted and separated using the water distillation method, utilizing the Clevenger device. The EO compounds were analyzed using a gas chromatograph coupled with a mass spectrometer (GC-MS). The evaluation of antimicrobial characteristics was carried out by determining the growth inhibition zone implementing the Agar method, the minimum inhibition concentration (MIC), and the minimum bactericidal/fungicidal concentration (MFC/MBC) utilizing liquid dilution culture.

**Results:**

The results indicated that the highest yield belonged to the EO of *D*. *kotschyi* at %2.6 (w/w). In this EO there were citral (%25.44), neral (%20.87), α-pinene (%14.48), trans-geranic acid methyl ester (%9.74), and D-limonene (%6.87). Moreover, *H*. *incanus* had the dominant compounds (-)-Spathulenol (%12.61), caryophyllene (%10.00), linolenic acid (%8.54), 1,8-cineole (%5.95), palmitic acid (%5.35), and α-cadinol (%5.17). The largest diameter of growth inhibition zone belonged to the *H*. *incanus* EO against the Gram-positive bacteria *S*. *pyogenes* (~17.67mm). The strongest inhibition activities in the form of growth inhibition diameter exhibited by the *D*. *kotschyi* EO were against the Gram-negative bacteria *S*. *paratyphi-A serotype* (~ 12 mm), *K*. *pneumoniae*, and *Sh*. *dysenteriae* (~ 11 mm) which was significant compared to the Gram-positive rifampin (~ 8 mm).

**Conclusions:**

It can be seen that these species are new chemotypes with special and novel chemical compounds which can potentially be used to manufacture natural antibiotics against some bacterial strains.

## 1. Introduction

Due to the excessive prescription of antibiotics and the increase in antibiotic resistance in bacteria the search for an alternative has become vital. Therefore, extensive research has been carried out about the potential use of bactericidal compounds in the EO of plants to control and treat illnesses [1, 2]. The use of Eos in pharmaceutical and food industries, alternative medicine, and herbal treatments are based on their antimicrobial effects [3, 4]. It has been proven that many Eos possess antimicrobial effects which makes them an environmentally friendly alternative to eliminate harmful bacteria and microbes [5, 6]. Researchers have proven that sometimes natural EOs have a better performance than artificial medicine. EOs have various mechanisms to fight against microorganisms such as preventing the building of cell walls and nucleic acids, inhibiting protein synthesis, and changing the cell membrane performance [7, 8]. The hydrophobe characteristic of the EOs allows them to penetrate the lipids in the cell membrane and increase its penetrability, disrupting all vital activities related to the cell membrane, the drainage of ions, vital compounds, and eventually apoptosis [9].

Nowadays, research on medicinal plants has been prioritized to facilitate the combination of various compounds to reach diverse biologic effects [10, 11]. Previous research has shown that the EO of many plants in the Lamiaceae family contains compounds of strong antimicrobial effects [12–15]. Of this family, the *Hymenocrater* type has 12 species in the world among which, 9 are bushes with beautiful colorful which grow in Iran. These plants grow in mountainous areas and 5 of these species are exclusive to Iran, with limited dispersal [16–18]. The species of this type are called “Arvaneh” flower or “Sheikh Ali” medicine in Iran and are used for culinary and medical purposes. These are used traditionally in the treatment of respiratory and heart diseases, skin allergies, scars, and as a nerve tonic, anti-inflammatory, air freshener, and mosquito repellant [19, 20]. In previous studies, various biologic activities have been reported for different species of *Hymenocrater* such as anti-oxidant [21, 22], anti-microbial [21, 23], anti-microbial [24], larvicidal [25], anti-diabetes, anti-Alzheimer’s, anti-obesity, and skin-repairing [26] effects. The *Hymenocrater incanus* Bunge species is an exclusive one in Iran which can be found in the west, south, and parts of central Iran [16]. The dominant compounds in the EO of this species are recorded as β-caryophyllene, 1,8-cineole, α-pinene, and β–pinene [27]. On the biologic activities of the *H*. *incanus* EO, there have only been reports of anti-microbial effects on some micro-organisms [28].

*Dracocephalum* is another type from the Lamiaceae family which has 186 different species throughout the world and 11 in Iran [17]. Five of these species are native to Iran and scattered in the north, west, east, and center of Iran [16]. The species of this type are called "Badarshaboo" in Farsi [29] *Dracocephalum* is a source of terpenoids such as Luteolin, Apigenin, Oleic acid, Ursolic acid, Neral, and Geraniol [30]. The EO of the plants of this type possess anti-oxidant, anti-bacterial, anti-bacterial, and antiseptic activities; they are also used to treat stomachache and bloating [31]. *Dracocephalum kotschyi* Boiss. is a species exclusive to the north, west, and center of Iran [32]. This plant is used for treating rheumatic pain, muscle building, wound healing, reducing blood fat, toning the immune system, and is regarded to have anticancer and anti-microbial effects in traditional medicine. The aroma and taste of the EO of these plants make them useful in many industries such as cosmetics, perfume manufacturing, drinks, ice-cream making, confectionery, food industries, etc. [33, 34]. The EO of this species is traditionally used as an anti-spasm agent [35]. Ethnobotanic monitoring reports in Iran indicate that this plant is known as “Semsa’” in Lorestan province and its leaves are traditionally used in cooking meat and fish, processing dairy products [41] (Ashrafi et al., 2017), and used as an antipyretic and sedative as well [36]. In Zanjan province, this plant is known as “Akhbash” and its leaves are infused to treat rheumatism [37]. Studies have shown that the EO of this species has pain-killing [38], antinociceptive [33], anti-spasm [39], anti-toxoplasma [40], anti-microbial [12] effects. The most important reported compounds of the EO of this species are monoterpenes such as α-pinene, limonene, geraniol, neral, and citral [29, 34, 39, 41–46] (7;; 5;. Plant EOs are affected by various factors such as genetics, evolution, environmental conditions, geographic changes, physiologic factors, and harvest time [44, 47]. Thus, plants that vary genetically and climatically and grow in different habitats can be considered strong sources to identify new chemotypes [28]. The difference in the chemical compounds in the EO of a species in various locations results in different anti-microbial activities [48].

Considering the traditional usage and the reported anti-microbial activities of the species of *Hymenocrater* and *Dracocephalum* types, and regarding the variety in the chemical compounds reported for *D*. *kotschyi* and *H*. *incanus*; it seems important to identify the chemotypes of these two species in various areas. Therefore, the present study was designed and carried out to identify the chemical compounds and evaluate and compare the antimicrobial activity of the EO of these two species for the first time in the natural habitats of Isfahan province in Iran.

## 2. Materials and methods

### 2.1. Species collection and identification

Initially, the natural habitats for *D*. *kotschyi* and *H*. *incanus* species were identified to be Totmach village (N 33˚ 41ʹ15” and E 51˚ 37ʹ 11” and 2077 m elevation) and Kamu village (N 33˚ 39ʹ42ʺ and E 51˚ 15ʹ 45ʺ and 2942 m elevation) in Isfahan province, Iran. Then, the flowering twigs of the species under study were collected randomly from various bases (70 bases in each spot) in May 2019. The specimens were moved to the laboratory after harvest and were spread on flat clean surfaces to dry. These species were identified and verified by a botanist and are recorded and kept under codes 1016 and 1017 in the herbarium of the Faculty of Natural Resources and Geoscience of the University of Kashan.

### 2.2. EO extraction and yield

Firstly, 100 g of the dry plant material of each harvest spot was ground to 1 cm pieces by an electric grinder. The plant materials were put into a 2-liter flask along with distilled water. The flask was coupled to a Clevenger device and after 2 hours the EO was extracted by the water distillation method. The EO gathered in the output pipes was separated using n-pentane. After that, the EO was completely separated from the water and purified using sodium sulphate. The extracted essence was poured into a dark and closed container and kept in a freezer (4°C). To determine the yield of the EO, the humidity percentage was calculated. This stage was repeated three times for the plant materials of each spot and the yield was reported as mean ± standard deviation [49].

### 2.3. Determining the chemical compounds of the EO

To determine the compounds of the EO a gas chromatograph connected to a mass spectrometer (GC-MS) model Agilent N-5973 made in the US, with an HP-5MS (%5 methyl phenyl siloxane, 30 m length, internal diameter 0.25 mm, absorbing material thickness 0.25 μm) was utilized. Regarding open temperature programming; first, the temperature reached 60°C in 5 mins. After that, the temperature was increased gradually with a 3°C/min rate until it reached 246°C. Helium was used as the carrier gas with a flow velocity of 1.5 mL/min. The ionization voltage was 70 eVs. The volume of the injected specimen was 1 μl with a 1.50 split and a detector temperature of 250°C. To calculate the retention index (RI) for the peaks, a mixture of aliphatic hydrocarbons (C8-C21) was injected into the GC system as per the analytical conditions mentioned above. The calculation and identification of the EO compounds were carried out using the linear retention indices and comparing them to the indices in references [50], and with the help of the mass spectrums of standard compounds and the data available in e-libraries.

### 2.4. Evaluating the anti-microbial activities of the EO

#### 2.4.1. Supply and culture of microbial variants

To evaluate the antimicrobial activity of the Eos, 12 standard microbial strains were obtained from the Iranian Research Organization for Science and Technology. These variants included four Gram-positive bacteria- *Bacillus subtilis* (ATCC 6633), *Staphylococcus aureus* (ATCC 29737), *Staphylococcus epidermidis* (CIP 81.55), and *Streptococcus pyogenes* (ATCC 19615)- five Gram-negative bacteria—*Escherichia coli* (ATCC 25922), *Klebsiella pneumonia* (ATCC 10031), *Pseudomonas aeruginosa* (ATCC 27853), *Salmonella paratyphi-A serotype* (ATCC 5702), and *Shigella dysenteriae* (PTCC 1188)-, two fungal variants- *Aspergillus brasiliensis* (ATCC 16404), and *Aspergillus niger* (ATCC 9029)-, and a yeast—*candida Albicans* (ATCC 16404). The bacterial variants were cultured in an Agar nutrient medium, whereas the fungi and yeast variants were cultured in the Sabouraud Dextrose Agar medium.

#### 2.4.2. Determining the growth inhibition zone by the Agar diffusion method

Various EOs were dissolved in Dimethyl sulfoxide (DMSO) to the concentration of 300 μg/mL. Plates of Mueller-Hinton Agar growth medium were supplied for the bacteria and Sabouraud Dextrose Agar for the fungi and the yeast. Microbial suspensions were prepared from the 24 h culture of various microbial variants with 0.5 McFarland turbidity, and 100 μl of each was cultured in similar growth media conditions. Utilizing Pasteur Pipettes, wells were made in the growth media with uniform distance and a 6 mm diameter. 10 μl EO was added to each well. To determine the sensitivity of the variants, the antibiotics rifampin and gentamicin (for the bacteria) and nystatin (for the fungi and the yeast) were used as a positive control and DMSO as a negative control. The plates impregnated with bacterial variants were incubated for 24 h in 37°C, plates impregnated with fungi variants for 72 h in 30°C and plates impregnated with the yeast for 48 h in 30°C in an incubator. Measuring the growth inhibition zone diameter was carried out using an antibiogram (in mm) [51] (Cockerill et al., n.d.). The tests were repeated for each EO sample and each variant, three times, and the growth inhibition zone diameter was reported in mean ± standard deviation.

#### 2.4.3. Determining the Minimum Inhibitory Concentration (MIC), and the Minimum Bactericidal/Fungicidal Concentration (MBC/MFC) with dilution in liquid growth media

A specific amount of every EO sample was weighed and the primary stock was prepared with a 4000 μg concentration. Then, concentrations of 1000, 2000, 500, 250, 125, 62.5 μg/mL were prepared from the primary concentration. A 96-well sterile microplate was utilized, and 95 μl of the broth growth medium, 5 μl of microbial suspension with 0.5McFarland, and 100 μl of each EO concentration were added to each microplate well. As a negative control, the growth medium was used instead of EOs, and as a positive control, antibiotic powders of gentamicin and rifampin (for the bacteria) and nystatin antibiotic powder (for the fungi and the yeast) were used instead of the EOs. The plates impregnated with bacterial variants were incubated at 37°C for 24 h, and the ones impregnated with yeast and fungi at 30°C for 48 and 72 h in an incubator. MIC was determined with regards to the turbidity and the change in the color in each of the microplate wells.

The agar dilution assay was used to determine the MICs for the fungal strains based on the protocol introduced by of [52]. The suitable amounts of EO at different concentrations (2000, 1000, 500, 250, 125, 62.5, 31.25 and 15.63 μg/mL) were added to SDA containing 50% (v/v) Tween 20. Nystatin powder was used as the positive control, and the negative control was the plate with SDA containing 50% (v/v) Tween 20 without any EO. The culture media were spot inoculated with 4 ml of spores (10^4^ spores /mL). The inoculated plates were incubated at 30°C for 72 h, the test was performed in triplicate for each essential oil, and the minimal concentration of the essential oil that inhibited the growth of the fungi was reported as the MIC.

After a 24 h heating period, 5 μl of each microplate well with no growth was injected into an agar nutrient medium to be incubated at 37°C for 24 h. Each concentration that did not exhibit growth had killed the microbe variant and was designated as the MFC/MBC [51] (Cockerill et al., n.d.). The tests were repeated three times for each EO and each variant.

### 2.5. Statistical analysis

To analyze the yield, the chemical compound, and microbial activity of the EOs, the Analysis of variance (ANOVA) was carried out. The difference between the data means was evaluated using the Duncan Post Hoc test in the significance level of 1.

## 3. Results and discussion

### 3.1. EO yield

The *D*. *kotschyi* EO (pale yellow) with a %2.6 (w/w) yield was significantly different from the *H*. *incanus* EO (dark yellow) with a %0.1 (w/w) yield. In previous studies, the highest yield from *D*. *kotschyi* was at %1.7 from Khansar area [43], %0.6 from Lorestan province [41], %0.45 from Zanjan [29], %0.24 from Fereydounshahr [42], and %0.2 from Daran [12]. Moreover, the yield of *H*. *incanus* was reported to be %0.6 in the Abadeh region [27] and %0.02 in the Daran area [28]. These quantities for medicinal herbs vary according to habitat and region because the metabolic activity of the plants fluctuates under the influence of environmental factors [53].

### 3.2. EO compounds

**Fig 1:**
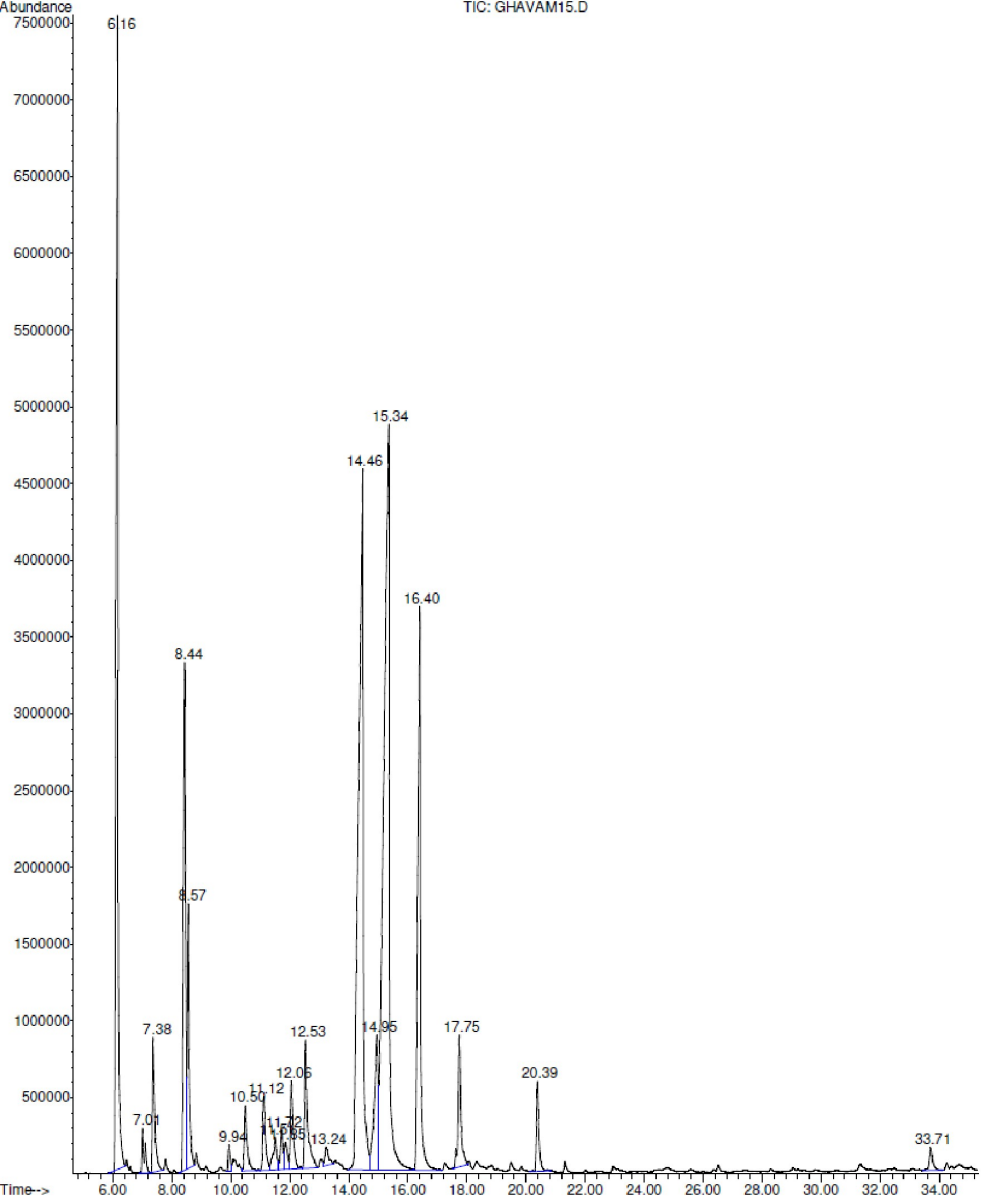
GC-MS chromatogram of essential oil of *D*. *kotschyi*.

**Fig 2:**
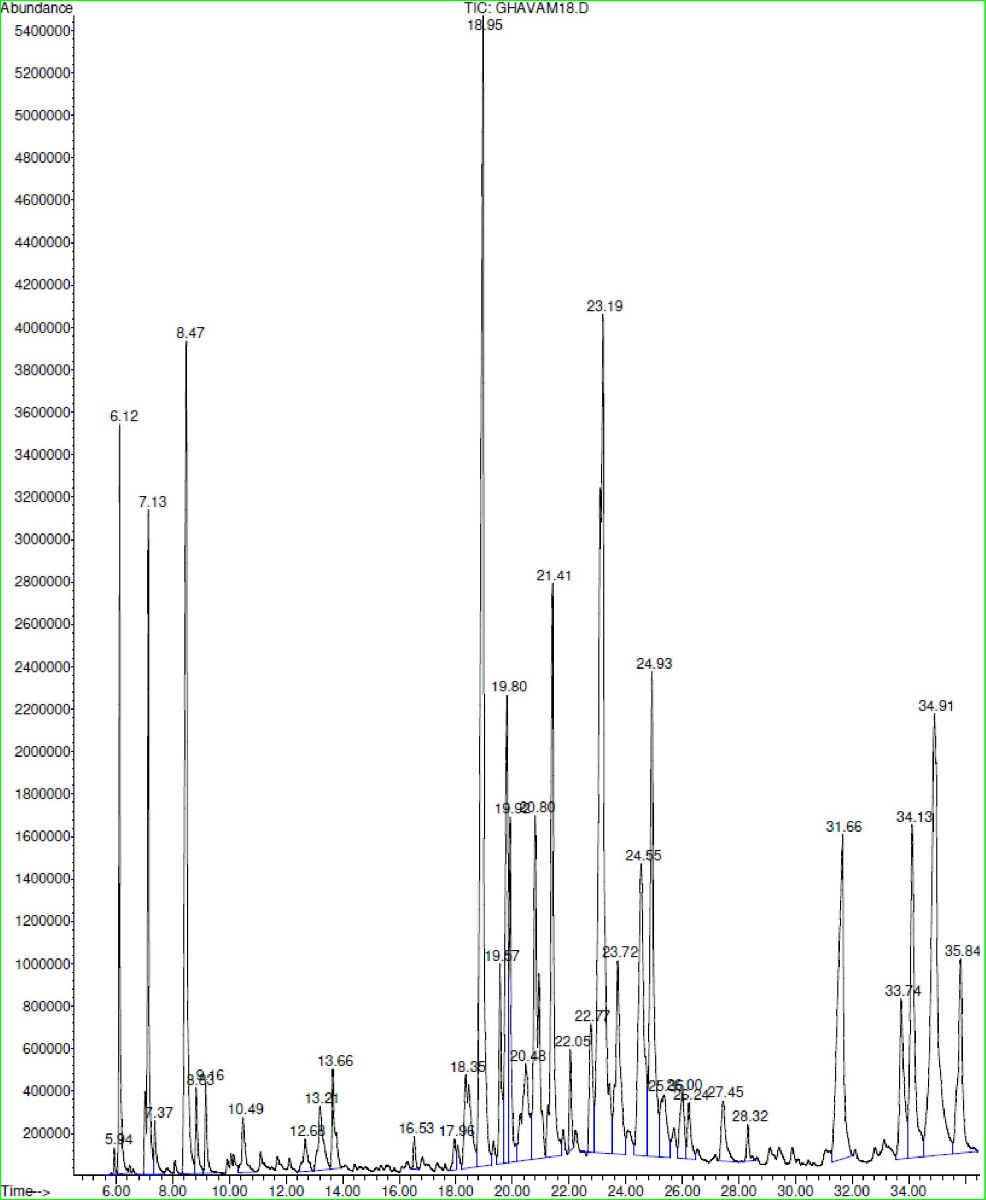
GC-MS chromatogram of essential oil of *H*. *incanus*.

The results of the analyses on the EOs revealed the existence of 54 different chemical compounds in the EO of the two species under study (Table 1) which the GC-MS chromatogram depicts in Figs 1 and 2. 36 of these compounds belonged to the *H*. *incanus* EO which is in accordance to the results of [27] with 35 compounds, and contradicts the results of [12] with 25. 21 various compounds were detected in the *D*. *kotschyi* EO which is in agreement with the findings of [43] with20 compounds, [12] with 21 compounds, and [42] with 23. Some previous studies reported the *D*. *kotschyi* EO compounds to be more than 40 which is in contrast with our findings [39, 41, 44].

**Table 1:** The chemical composition of essential oil of *H*. *incanus* and *D*. *kotschyi*.

no.	Compound	RI^c^	RI^n^	*Dracocephalum kotschyi* Boiss	*Hymenocrater incanus* Bunge	Molecular formula
1	α-Thujene	873.891	873	-	0.10±0.01 ^a^	C_10_H_16_
2	α-Pinene	882.758	882	14.48±0.02 ^a^	3.03±0.00 ^b^	C_10_H_16_
3	Sabinene	917.880	916	0.71±0.00 ^a^	-	C_10_H_16_
4	β -Pinene	921.854	920	-	3.39±0.01 ^a^	C_10_H_16_
5	β -Myrcene	929.801	930	1.95±0.00 ^a^	0.39±0.00 ^b^	C_10_H_16_
6	D-Limonene	965.231	965	6.87±0.02 ^a^	-	C_10_H_16_
7	1,8-Cineole	966.225	966	-	5.95±0.00 ^a^	C_10_H_18_O
8	trans-β-Ocimene	969.536	970	3.14±0.00 ^a^	-	C_10_H_16_
9	β-Ocimene	978.145	978	-	0.55±0.00 ^a^	C_10_H_16_
10	γ-Terpinene	987.072	986	-	0.54±0.00 ^a^	C_10_H_16_
11	α-Terpinolene	1011.904	1012	0.32±0.00 ^a^	-	C_10_H_16_
12	Linalool	1026.455	1026	1.22±0.03 ^a^	0.55±0.00 ^b^	C_10_H_18_O
13	α-Campholenal	1043.121	1042	1.22±0.00 ^a^	-	C_10_H_18_O
14	6-Octenal, 7-methyl-3-methylene-	1053.439	1053	0.80±0.00 ^a^	-	C_10_H_18_O
15	trans-Chrysanthemal	1058.994	1060	0.63±0.00 ^a^	-	C_10_H_16_O
16	trans-Verbenol	1062.433	1063	0.51±0.00 ^a^	-	C_10_H_16_O
17	Isoneral	1067.989	1069	1.55±0.00 ^a^	-	C_10_H_16_O
18	Isogeranial	1080.423	1080	2.52±0.02 ^a^	-	C_10_H_16_O
19	(-)-4-Terpineol	1084.391	1085	-	0.41±0.00 ^a^	C_10_H_18_O
20	α-Terpineol	1098.412	1098	-	0.93±0.00 ^a^	C_10_H_18_O
21	(-)-α-Terpineol	1099.206	1099	0.42±0.00 ^a^	-	C_10_H_18_O
22	cis-3-Hexenyl-α-methylbutyrate	1109.375	1110	-	0.84±0.00 ^a^	C_11_H_20_O_2_
23	Neral	1128.605	1129	20.87±0.04 ^a^	-	C_10_H_18_O
24	Geraniol	1140.384	1140	3.42±0.00 ^a^	-	C_10_H_18_O
25	Citral	1149.519	1150	25.44±0.00 ^a^	-	C_10_H_18_O
26	1,5,5-Trimethyl-6-methylene-cyclohexene	1174.038	1173	-	0.19±0.01 ^a^	C_10_H_16_
27	trans-Geranic acid methyl ester	1175.240	1175	9.74±0.00 ^a^	-	C_11_H_18_O_2_
28	Geranyl acetate	1207.582	1208	2.28±0.00 ^a^	-	C_12_H_20_O_2_
29	β-Damascenone	1212.559	1213	-	0.24±0.00 ^a^	C_13_H_18_O
30	α-Longipinene	1221.800	1222	-	1.75±0.00 ^a^	C_15_H_24_
31	Caryophyllene	1236.018	1237	-	10.00±0.00 ^a^	C_15_H_24_
32	cis-β-Farnesene	1250.473	1251	-	1.37±0.00 ^a^	C_15_H_24_
33	α-Humulene	1256.161	1256	-	3.79±0.00 ^a^	C_15_H_24_
34	Alloaromadendrene	1259.002	1259	-	3.31±0.00 ^a^	C_15_H_24_
35	Germacrene D	1270.142	1271	1.41±0.00 ^a^	-	C_15_H_24_
36	Bicyclo[4.4.0]dec-1-ene, 2-isopropyl-5-methyl-9-methylene-	1272.274	1272	-	1.90±0.00 ^a^	C_15_H_24_
37	β-Cyclogermacrane	1279.857	1280	-	4.18±0.02 ^a^	C_15_H_24_
38	δ-cadinene	1294.549	1295	-	4.83±0.00 ^a^	C_15_H_24_
39	Chysin A	1309.443	1309	*-*	*0*.59±0.00 ^a^	C_9_H_15_NO_2_
40	cis-3-Hexenyl benzoate	1327.118	1328	-	1.34±0.00 ^a^	C_13_H_16_O_2_
41	(-)-Spathulenol	1337.288	1338	-	12.61±0.01 ^a^	C_15_H_24_O
42	Humulene epoxide II	1350.121	1350	-	2.60±0.00 ^a^	C_15_H_24_O
43	τ-Muurolol	1370.217	1570		4.42±0.00 ^a^	C_15_H_26_O
44	α-Cadinol	1379.418	1379	-	5.17±0.00 ^a^	C_15_H_26_O
45	β-Copaen-4α-ol	1405.793	1406	-	0.80±0.00 ^a^	C_15_H_24_O
46	Cypera-2,4-diene	1411.586	1412	-	0.55±0.00 ^a^	C_15_H_22_
47	Myristic acid	1442.065	1443	-	0.77±0.00 ^a^	C_14_H_28_O_2_
48	2-Pentadecanone, 6,10,14-trimethyl-	1463.979	1465	-	0.27±0.00 ^a^	C_18_H_36_O
49	Palmitic acid	1550.263	1550	-	5.35±0.01 ^a^	C_16_H_32_O_2_
50	6-Methyl-4,6-bis(4-methylpent-3-en-1-yl)cyclohexa-1,3-dienecarbaldehyde	1604.709	1603	0.49±0.00 ^a^	-	C_20_H_30_O
51	Phytol	1605.263	1607	-	1.98±0.00 ^a^	C_20_H_40_O
52	Triamterene	1616.066	1616	-	4.23±0.00 ^a^	C_12_H_11_N_7_
53	Linolenic acid	1637.950	1638	-	8.54±0.00 ^a^	C_18_H_30_O_2_
54	Silane, dimethyl(2-chlorophenoxy)docosyloxy-	1663.434	1663	-	2.58±0.00 ^a^	C_26_H_47_ClO_2_Si
	Total			100	100	
	Monoterpenes hydrocarbons			27.47	8.19	
	Oxygenated monoterpenes			58.62	7.84	
	Sesquiterpenes hydrocarbons			1.41	31.13	
	Oxygenated sesquiterpenes			0	25.6	
	Others			12.51	27.28	

RI ^n^ refers to the retention index identified by database NIST 014; RI^c^ refers to the retention index calculated from the retention time relative to that of C8 –C40 n-alkanes; Values with different letters are statistically different (Duncan, p ≤ 0.05)

Oxygenated monoterpenes constituted %58.62 of the *D*. *kotschyi* EO compounds which agrees with the findings of [28, 43, 44]. The EO of *H*. *incanus* contained %31.13 sesquiterpenes hydrocarbons as the dominant group of chemical compounds. [28] found nonterpenoids (others) as the dominant compounds of this EO at %48.98 which contradicts our findings. The diversity of phytochemical compounds in various chemotypes is impacted by factors such as environmental, soil, and seasonal conditions [54].

The results of variance analysis indicated that a significant difference existed among the means of the amounts of various components from the EOs under study (P≤0.01). The dominant and main compounds in the EO of *D*. *kotschyi* were citral (%25.44), neral (%20.87), α-pinene (%14.48), trans-geranic acid methyl ester (%9.74), and D-limonene (%6.87). In some previous studies, citral at %12.08 [41] and %19.63–12.49 [55] was reported to be the primary compound of *D*. *kotschyi* which agrees with our findings. Moreover, previous studies reported neral as the third main compound in the EO of this species at %20.9 in Mazandaran province, %11.58 in Ardebil province, and %11.25 in Isfahan province [28, 44, 56]. The α-pinene compound in the EO of this species was reported by [28, 41] to be the dominant compound at %10.43 and %13.66 respectively, which contrasts our present findings. Trans-Geranic acid methyl ester has never been reported in previous studies on this plant, which makes our study the first to report the presence of these compounds in this EO; this could be the result of chemotypical variations. Some previous studies reported D-limonene as the second dominant compound for this EO at %19.96 [43], and %46.79 [29]. The reason for the difference between our findings and those of previous studies can be due to ecologic conditions, environmental conditions, the growth stage of the plant, harvest time, EO extraction methods, or even storage conditions [57].

The dominant compounds of the *H*. *incanus* EO were (-)-Spathulenol (%12.61), caryophyllene (%10.00), linolenic acid (%8.54), 1,8-cineole (%5.95), palmitic acid (%5.35), and α-cadinol (%5.17). (-)-spathulenol has been reported as the stereoisomer spathulenol in previous studies at %2.7 [27]. Thus, the existence and dominance of this compound in this EO in our study is a specific characteristic that has not been reported before. In previous reports on the compounds of the EO of this species, caryophyllene was mentioned as the dominant compound (%17.6), and as a peripheral compound (%3.68) [27, 28]. The existence of acidic compounds such as oleic acid (%23.53), and palmitic acid (%28.23) as the second and third dominant compounds of this EO was previously reported [28]. The compound 1,8-cineole was reported as the first compound at %16.9 by [27], and as a peripheral compound at %0.22 by [28] which contradicts the findings of the present study. Similarly, [58] reported the fifth dominant compound of this EO to be α-cadinol at %7.66. The difference in type and quantity of the chemical compounds in the EO indicates the unique properties of the species in the area under study which can result from a difference in the environmental conditions of the habitat and imply the existence of a chemotype of this species [28, 59].

### 3.3. Antimicrobial activity

The ANOVA results showed that there is a significant difference between the diameter of the growth inhibition zone for the EOs under study and the Gram-positive antibiotics against the microbial variants (P≤0.01) (Table 2). The biggest growth inhibition zone diameter belonged to the *H*. *incanus* EO against the Gram-positive bacteria *S*. *pyogenes* (~17.67 mm) which can be called an acceptable antibacterial activity compared to the antibiotics rifampin (~21 mm) and gentamicin (~ 32 mm). These bacteria are responsible for the most common bacterial pharyngitis [60], scarlet fever, and impetigo. The present study is the first report of the inhibitory power of the *H*. *incanus* EO against *S*. *pyogenes* and can introduce a potential natural tool against this bacteria. The biological activity of the Eos depends on their chemical compounds [61]. Due to the hydrophobic nature of the effective compounds in Eos, they cannot penetrate and access the active points inside Gram-negative bacteria. Therefore, Gram-positive bacteria usually show more vulnerability to these Eos [62]. The dominance of sesquiterpenes such as (-)-spathulenol, caryophyllene, and α-cadinol, the monoterpene 1,8-cineole, and acidic compounds like linolenic acid and palmitic acid seems to be the main reason for this antibacterial activity. [63] have also attributed the antimicrobial activity of the *H*. *incanus* EO to the dominance of oxygenated sesquiterpenes. The antibacterial activity of spathulenol has been proven [64]. Similarly, [65] has reported that the antibacterial activity of the *H*. *longiflorus* was due to the marginal or rare existence of antibacterial compounds, specifically 1,8-cineole. The antimicrobial activity of caryophyllene against Gram-positive bacteria has been proven [66]. [67] have attributed the strong antibacterial activity of the *Calendula officinalis* L. to the impressive amounts of α-cadinol. The antimicrobial activity of the acidic compounds against many microorganisms has been proven [28, 68]. Moreover, the synergy resulting from the diversity among the main and peripheral EO compounds in their antimicrobial activities must be considered [28]. Therefore, compounds with lower percentages such as α-pinene, β–pinene, linalool, cis-β-farnesene, α-humulene, δ-cadinene, and phytol can be considered as other effective factors in the antibacterial activity. In many studies, the activity against *S*. *pyogenes* is attributed to the presence of α-pinene and linalool in the EOs [15]. Strong antibacterial activity is reported for phytol [69]. The antibacterial and anti-biofilm effects of α-humulene extracted from plants have been proven [70]. The inhibitory activity of β–pinene against some Streptococcus variants has been recorded.

**Table 2:** Diameter of growth inhibition zone, Minimal inhibitory concentrations (MIC) and Minimal bactericidal (MFC) concentrations of essential oil of *H*. *incanus* and *D*. *kotschyi* and referent antibiotics.

Standard microbial strains	*D*. *kotschyi*			*H*. *incanus*			Nystatin		Gentamicin		Rifampin	
	IZ	MIC	MBC	IZ	MIC	MBC	IZ	MIC	IZ	MIC	IZ	MIC
*Bacillus subtilis* (ATCC 6633)	―	2000±0.00 ^b^	4000±0.00 ^a^	―	2000±0.00 ^b^	4000±0.00 ^a^	NA	NA	30±0.00 ^c^	3.90300±.00 ^e^	19±0.00 ^d^	31.25±0.00 ^c^
*Escherichia coli* (ATCC 25922)	―	2000±0.00 ^a^	2000±0.00 ^a^	―	2000±0.00 ^a^	2000±0.00 ^a^	NA	NA	20±0.00 ^b^	3.90±0.00 ^d^	11±0.00 ^c^	3.90±0.00 ^d^
*Klebsiella pneumonia* (ATCC 10031)	11±0.20	2000±0.00 ^a^	2000±0.00 ^a^	―	2000±0.00 ^a^	2000±0.00 ^a^	NA	NA	17±0.00 ^b^	3.90±0.00 ^d^	8±0.00 ^c^	15.63±0.00 ^b^
*pseudomonas aeruginosa* (ATCC 27853)	―	2000±0.00 ^a^	2000±0.00 ^a^	―	2000±0.00 ^a^	2000±0.00 ^a^	NA	NA	20±0.00 ^c^	7.81±0.00 ^d^	―	31.25±0.00 ^b^
*Salmonella paratyphi-A serotype* (ATCC 5702)	12±0.00 ^d^	2000±0.00 ^a^	2000±0.00 ^a^	―	2000±0.00 ^a^	2000±0.00 ^a^	NA	NA	18±0.00 ^b^	3.90±0.00 ^e^	8±0.00 ^a^	15.63±0.00 ^c^
*Shigella dysenteriae* (PTCC 1188)	11±0.50 ^d^	2000±0.00 ^a^	2000±0.00 ^a^	―	2000±0.00 ^a^	2000±0.00 ^a^	NA	NA	17±0.00 ^b^	3.90±0.00 ^f^	9±0.00 ^e^	15.36±0.00 ^c^
*Staphylococcus aureus* (ATCC 29737)	―	2000±0.00 ^a^	4000±0.00 ^a^	―	2000±0.00 ^a^	4000±0.00 ^a^	NA	NA	27±0.00 ^b^	1.95±0.00 ^d^	21±0.00 ^c^	31.25±0.00 ^b^
*Staphylococcus epidermidis (CIP 81*.*55)*	―	2000±0.00 ^a^	2000±0.00 ^a^	―	2000±0.00 ^a^	2000±0.00 ^a^	NA	NA	45±0.00 ^b^	1.95±0.00 ^d^	27±0.00 ^c^	1.95±0.00 ^d^
*Streptococcus pyogenes* ATCC 19615	―	250±0.00 ^b^	2000±0.00 ^a^	17±0.50 ^e^	2000±0.00 ^a^	2000±0.00 ^a^	NA	NA	32±0.00 ^c^	0.975±0.00 ^f^	21±0.00 ^d^	0.975±0.00 ^a^

IZ: The diameters of growth inhibition zone includes the diameters of disks (6 mm); ND: not determined; NA: no activity; Values with different letters are statistically different (Duncan, p≤0.05).

The strongest inhibitory activity of the *D*. *kotschyi* EO was against the Gram-negative bacteria *S*. *paratyphi-A serotype* with the growth inhibition zone diameter of ~12 mm, comparative to rifampin (~8 mm) and gentamicin (~18 mm). Similarly, [41] reported a growth inhibition zone diameter of 30 mm for the *D*. *kotschyi* EO. Salmonellas cause typhoid fever, gastroenteritis, and sepsis in humans and can be contracted by direct contact, or food and water contaminated by human or animal excrement [71]. The difference in the antibacterial activity of the EO of a species from various habitats results from the effect of the geographical location of the plant on the type and percentage of its phytochemical compounds [1]. This strong activity seems to be mainly due to the dominance of oxygenated monoterpenes such as citral and neral, hydrocarbon monoterpenes like α-pinene and D-limonene, and acidic compound trans-geranic acid methyl ester. The molecular structure of terpenes makes them hydrophobe which enables them to precipitate on the lipophilic plasma membrane of microorganisms. This increases the permeability of the membrane which in turn causes the loss of vital electrolytes [72]. The antibacterial effects of citral and neral are proven [73]. The inhibitory effect of α-pinene has been reported against some salmonella variants [74]. Moreover, minor and peripheral compounds such as trans-β-ocimene, linalool, β–myrcene, geraniol, germacrene D, and geranyl acetate could be effective in this activity. The inhibitory effect of myrcene [74], linalool [75]. and EOs containing sabinene and trans-β-ocimene [76] against some Salmonella variants has been recorded.

Another strong inhibitory activity of the *D*. *kotschyi* EO was producing a growth inhibition zone diameter of about 11 mm against Gram-negative bacteria *K*. *pneumoniae* and *Sh*. *dysenteriae* which was extremely significant and impressive compared to the positive controls rifampin (~8 mm) and gentamicin (~17 mm). In previous studies the growth inhibition zone diameter of this EO against *K*. *pneumoniae* was reported to be about 10 mm by [52], 18 mm by [41], and 10 mm by [46]. Furthermore, the growth inhibition zone diameter of this EO against *Sh*. *dysenteriae* has been reported to be about (9.50±0.50mm) [58]. Klebsiella pneumoniae is an opportunistic bacterium in clinical settings and could cause nosocomial infections such as ureteral, respiratory, and blood flow infections, especially in ICU patients [77]. There have been reports of inhibitory activities against *K*. *pneumoniae* for geraniol [78], myrcene, and α-pinene [74], linalool [79], and Limonene [80]. Shigella is one of the most important causes of intestine infections and diarrhea [81]. Dysentery caused by *Shigella* is usually serious and can lead to death if not treated on time [82]. The effect of some compounds such as geraniol and α-pinene against some *Shigella* sp. has been proven [83, 84].

The *D*. *kotschyi* EO had a growth inhibition zone diameter of about 8 mm against the yeast *C*. *albicans* which was weaker compared to nystatin (~33 mm) which contradicts the results reached by [58] for this EO against *C*. *albicans* (16.33±0.58). *C*. *albicans* is an opportunistic fungal pathogen that dwells in the gut and urethra of %70 of humans, and %75 of women suffer from a Candida infection at least once in their lives. This yeast will turn into an opportunistic pathogen in immunodeficient patients, people with impaired immune systems, or even healthy people [85, 86] and cause reproductory, skin, mouth, and blood infections [6, 87]. [88] have reported strong activity for linalool and citral against *C*. *albicans*. (da Silva et al., 2008) [89, 90] (Leite et al., 2014) have shown significant in vitro activity for citral against *C*. *albicans*. A growth inhibition zone diameter of (~11 mm) against *C*. *albicans* was recorded for a pure compound of α-pinene. The inhibitory effect of the EOs containing germacrene D against this yeast has been reported [91] (Mevy et al., 2007). The anti-Candida effects of Limonene [80], linalool [92], and geraniol [93, 94] have been proven.

On the other hand, the findings of the minimum inhibitory or killing concentration using the dilution method in liquid growth media indicated that the value for MBC and MIC for *D*. *kotschyi* ad *H*. *incanus* EOs against all microorganisms under study fluctuated between 1000 and 4000 μg/mL which implies a weak performance compared to the positive control antibiotics (Table 3). The least value for minimum inhibitory concentration belongs to the *D*. *kotschyi* EO against *S*. *pyogenes* at 250 μg/mL which was quite weak compared to the positive controls rifampin and gentamicin (MIC = 0.975 μg/mL).

**Table 3:** Diameter of growth inhibition zone, Minimal inhibitory concentrations (MIC) and Minimal fungicidal (MFC) concentrations of essential oil of *H*. *incanus* and *D*. *kotschyi* and referent antibiotics.

Standard microbial strains	*D*. *kotschyi*			*H*. *incanus*			Nystatin		Gentamicin		Rifampin	
	IZ	MIC	MBC	IZ	MIC	MBC	IZ	MIC	IZ	MIC	IZ	MIC
*Aspergillus brasiliensis* (ATCC 16404)	―	1000±0.00 ^b^	1000±0.00 ^b^	―	2000±0.00 ^a^	2000±0.00 ^a^	30±0.00^c^	31.2±0.00 ^c^	NA	NA	NA	NA
*Aspergillus niger* (ATCC 9029)	-	1000±0.00 ^b^	1000±0.00 ^b^	―	2000±0.00 ^a^	2000±0.00 ^a^	27±0.00 ^d^	31.2±0.00 ^c^	NA	NA	NA	NA
*Candida albicans* (ATCC 10231)	8±0.50 ^d^	2000±0.00 ^a^	2000±0.00 ^a^		2000±0.00 ^a^	2000±0.00 ^a^	125±0.00 ^b^	33±0.00 ^c^	NA	NA	NA	NA

IZ: The diameters of growth inhibition zone includes the diameters of disks (6 mm); ND: not determined; NA: no activity; Values with different letters are statistically different (Duncan, p≤0.05).

There has not been any report of the effect of this EO against *S*. *pyogenes* which makes our report the first to do so. It seems that the dominance of sesquiterpenes in the *D*. *kotschyi* EO has a vital role in this inhibitory activity compared to the *H*. *incanus* EO. Similarly, [58] deemed the dominance of sesquiterpenes as the main factor in the inhibitory activity of *Rosa damascene* Mill EO against this bacteria. The strong antimicrobial activity of sesquiterpenes is proven [95].

## 4. Conclusion

The results show that the *D*. *kotschyi* and *H*. *incanus* EOs had significant differences in yield, chemical compounds, and antimicrobial characteristics. The dominance of oxygenated sesquiterpene compounds in the *D*. *kotschyi* EO such as citral and neral and the acidic compound trans-geranic acid methyl ester for the first time caused antibacterial effects which were stronger than rifampin against Gram-negative bacteria *S*. *paratyphi-A serotyp*, *K*. *pneumoniae*, *and Sh*. *dysenteriae*. In the *H*. *incanus* EO, the dominance of hydrocarbon sesquiterpenes such as (-)-Spathulenol and caryophyllene, and acidic compounds like linolenic acid and palmitic acid are the unique attributes linked to the conditions of the growth media, causing the largest diameter of growth inhibition zone against Gram-positive bacteria, *S*. *pyogenes*. Therefore, the present study confirms the effects of habitat on the synthesis of chemical compounds in the EO of these two species as new chemotypes which can be a potential natural antibiotic against some infectious diseases. Nevertheless, more research for clinical applications is needed in the future.

## Supporting information

S1 File (RAR)

## List of abbreviations

EOs: Essential oils
GC-MS: Gas chromatography–mass spectrometry
μg: Microgram
mL: Miligram
DMSO: dimethyl-sulfoxide
MFC: Minimum Fungicidal Concentration
MBC: Minimum Bactericidal Concentration
MIC: Minimal inhibitory concentrations
RI: Retention Index
